# Transplantation of ATP7B–Transduced Bone Marrow Mesenchymal Stem Cells Decreases Copper Overload in Rats

**DOI:** 10.1371/journal.pone.0111425

**Published:** 2014-11-06

**Authors:** Shenglin Chen, Cunhua Shao, Tianfu Dong, Hao Chai, Xinkui Xiong, Daoyi Sun, Long Zhang, Yue Yu, Ping Wang, Feng Cheng

**Affiliations:** 1 Department of Hepatobiliary Surgery Ward of General Surgery, The Affiliated Wuhu No. 2 People's Hospital of Wannan Medical College, Wuhu, Anhui Province, China; 2 Department of Hepatobiliary Surgery, Dongying People's Hospital, Dongying, Shandong Province, China; 3 Liver Transplantation Center, First Affiliated Hospital of Nanjing Medical University, Nanjing, Jiangsu Province, China; 4 Key Laboratory of Living Donor Liver Transplantation, Ministry of Public Health, Nanjing, Jiangsu Province, China; University of Louisville, United States of America

## Abstract

**Background:**

Recent studies have demonstrated that transplantation of ATP7B-transduced hepatocytes ameliorates disease progression in LEC (Long-Evans Cinnamon) rats, a model of Wilson's disease (WD). However, the inability of transplanted cells to proliferate in a normal liver hampers long-term treatment. In the current study, we investigated whether transplantation of ATP7B-transduced bone marrow mesenchymal stem cells (BM-MSCs) could decrease copper overload in LEC rats.

**Materials and Methods:**

The livers of LEC rats were preconditioned with radiation (RT) and/or ischemia-reperfusion (IRP) before portal vein infusion of ATP7B-transduced MSCs (MSCs^ATP7B^). The volumes of MSCs^ATP7B^ or saline injected as controls were identical. The expression of ATP7B was analyzed by real-time quantitative polymerase chain reaction (RT-PCR) at 4, 12 and 24 weeks post-transplantation. MSC^ATP7B^ repopulation, liver copper concentrations, serum ceruloplasmin levels, and alanine transaminase (ALT) and aspartate transaminase (AST) levels were also analyzed at each time-point post-transplantation.

**Results:**

IRP-plus-RT preconditioning was the most effective strategy for enhancing the engraftment and repopulation of transplanted MSCs^ATP7B^. This strategy resulted in higher ATP7B expression and serum ceruloplasmin, and lower copper concentration in this doubly preconditioned group compared with the saline control group, the IRP group, and the RT group at all three time-points post-transplantation (*p*<0.05 for all). Moreover, 24 weeks post-transplantation, the levels of ALT and AST in the IRP group, the RT group, and the IRP-plus-RT group were all significantly decreased compared to those of the saline group (*p*<0.05 compared with the IRP group and RT group, *p*<0.01 compared with IRP-plus-RT group); ALT and AST levels were significantly lower in the IRP-plus-RT group compared to either the IRP group or the RT group (*p*<0.01 and *p*<0.05. respectively).

**Conclusions:**

These results demonstrate that transplantation of MSCs^ATP7B^ into IRP-plus-RT preconditioned LEC rats decreased copper overload and was associated with an increase in MSC engraftment and repopulation.

## Introduction

Wilson's disease (WD), also known as hepatolenticular degeneration, is an autosomal recessive inherited disease that is caused by a mutation in the ATP7B gene and is characterized by impaired biliary copper excretion and lack of ceruloplasmin synthesis [1]. This leads to copper accumulation in the liver, brain, kidney, and other organs [2]. Because WD is a monogenic hereditary disease, gene therapy could potentially reverse the defect. Many genetic diseases could theoretically be cured by transplantation of normal cells. However, in order to have a beneficial clinical impact, transplanted cells must effectively engraft in the host, and maintain their proliferative capacity. To this point in time, the most effective treatment to correct copper metabolism and prevent the progression of WD has been orthotopic liver transplantation [3], [4]. However, shortage of quality donor organs, low hepatocyte viability (only 30% of hepatocytes survive transplantation) and post-transplantation complications have hampered more widespread use of this clinical therapy [4]–[6]. Therefore, cell-based therapy is emerging as a potential tool in regenerative medicine [7]. This is the case because of the ease of retroviral vector–mediated integration of exogenous genes into chromosomes of target cells, with the outcome of long-term gene expression. However, retroviral vectors cannot efficiently transfect non-dividing cells. In contrast, lentiviral vectors can transfect both dividing cells as well as non-dividing cells such as hepatocytes. In addition, they can accommodate large inserts—up to 10 kb—and the inserted fragments integrate into target-cell chromosomes. The lentiviral vectors are composed of 3 basic genetic structures: gag, pol, and env genes, four auxiliary genes—vif, vpr, nef, and vpu and two regulatory genes—tat and rev [8]. Because of the presence of a transcription promoter, more efficient, long-term gene expression, and lower level of induced immune response, lentiviral vectors are superior to both retroviral and adenoviral vectors [9]–[11].

Bone marrow mesenchymal stem cells (BM-MSCs) are a type of adult stem cell. They have attracted much attention due to their high capacity for self-renewal, multipotent differentiation potential, ready availability, and low occurrence of adverse reactions post-transplantation. They are being studied for a variety of clinical applications [12]. Studies have found that BM-MSCs can differentiation into osteoblasts [13], chondrocytes [14], adipocytes [15], neurons [16], cardiomyocytes [17], and hepatocytes [18]. When co-cultured with hepatocytes, they promoted growth of the hepatocytes. When transplanted into liver, they likewise promoted the growth of hepatocytes *in vivo*, and accelerated restoration of function in damaged livers [19]. Studies have also shown that BM-MSCs migrate to a damaged liver and promote liver regeneration [20]. More importantly, because of the ease of transfection of BM-MSCs with exogenous genes, they can be used as vectors of these exogenous genes [21]. A number of studies have shown the therapeutic effects of BM-MSCs in animal models of acute liver failure and liver fibrosis. However, there have been few such studies in congenital hepatic metabolic diseases [22].

LEC (Long-Evans Cinnamon) rats have a deletion mutation of 900 bp in the 3′-terminal region of Atp7b, the rat homologue of the human WD gene, ATP7B [23], [24]. This deletion leads to hepatic copper accumulation in the LEC rat [25]. Therefore, the LEC rat provides an excellent animal model for human copper overload [26]. The aim of the present study was to transfect the ATP7B gene into BM-MSCs, and determine the effects of transplantation of those cells on copper overload in LEC rats preconditioned by ischemia-reperfusion (IRP) and/or radiation (RT).

## Materials and Methods

### Animal Care

LEC rats were kindly provided by S. Gupta (Albert Einstein University College of Medicine, NY, USA). All animals used in this study were bred and maintained in individually ventilated cages in the animal facilities at Nanjing Medical University under controlled temperature (23±2°C) and humidity (55±10%) under 12 hour light/12 hour dark cycle, with tap water and food available *ad libitum*. The experimental protocol was approved by Nanjing Medical University/Institutional Animal Care and Use Committee (NJMU/IACUC). Ninety healthy two month old LEC rats were used in this research, they were randomly divided into 5 groups:, group a were injected with saline, group b were injected with MSCs^ATP7B^, groups c, d and e had livers preconditioned with ischemia-reperfusion (IRP), radiation (RT) and ischemia-reperfusion plus radiation (IRP-plus-RT), respectively before injection with MSCs^ATP7B^.

### Isolation of BM-MSCs

BM-MSCs were isolated from healthy 3–4 week old LEC rats using a previously described protocol [27]. The rats were sacrificed, soaked in 75% ethanol for 5 min, with subsequent isolation of femurs and tibias under sterile conditions. Samples were placed in phosphate buffered saline (PBS) containing 100 U/mL penicillin, and 100 µg/mL streptomycin (GIBCO, Grand Island, NY, USA). The ends of the diaphyses were removed, and the bone marrow cavity flushed with PBS. The cell suspension was filtered through nylon mesh and centrifuged at 300×g for 5 min at room temperature. The obtained cells were resuspended in SD rat mesenchymal stem cell growth medium (Cyagen Biosciences, Guangzhou, China) and cultured in 25 cm^2^ plastic culture flasks (Corning Incorporated, Corning, NY, USA) at 37°C under 5% CO_2_. Fourth-to-eighth passage BM-MSCs were used in this study.

### Immunophenotyping of BM-MSCs

Flow cytometric analysis of the surface markers of BM-MSCs were performed on a BD FACS Calibur flow cytometer (BD Biosciences, San Jose, CA, USA) by a method described in our previous work [27]. The fifth passage of BM-MSCs were harvested and re-suspended in 0.1 mL PBS. The cell suspensions were incubated with the following antibodies for 15 min at 4°C: anti-rat CD29 conjugated with phycoerthrin (PE), anti-rat CD90 conjugated with PE, anti-rat CD45 conjugated with fluorescein isothiocyanate (FITC), anti-rat CD11b/c conjugated with PerCP-eFluor710 (eBioscience, San Diego, CA, USA). PE-conjugated mouse IgG2a, PE-conjugated Armenian Hamster IgG, FITC-conjugated mouse IgG1, and PerCP-e710 mouse IgG2a were used as an isotype-matched control.

### Lentiviral vectors

All plasmids used in this study were kindly provided by Professor Duanqing Pei, Drug Discovery Pipeline Group, Guangzhou Institutes of Biomedicine and Health, Chinese Academy of Sciences. In order to produce ATP7B lentiviral particles, HEK293T cells were co-transfected with pRRL.PPT.SF.ATP7B.i2GFPBsd.pre and two packaging plasmids, psPAX2 and pMD2.G through calcium phosphate-mediated transient transfection, as previously described [28]. HEK293T cells were also co-transfected with pRRL.PPT.SF.i2GFPpre and the two packaging plasmids mentioned above as negative controls. Viral supernatants were harvested 48 hours post-transfection.

### Transduction of BM-MSCs in Cell Culture

Fourth-or-fifth passage BM-MSCs were used for transduction. BM-MSCs 70–80% confluent, were digested with trypsin, and then seeded into six-well plates at a density of 2–3×10^5^ cells/well. Twenty-four hours after subculture, the medium was discarded, cells were gently rinsed with PBS and treated with 1 mL viral supernatants and 8 µg/mL polybrene per well for 4 h. Subsequently, 1 mL conventional BM-MSC culture medium (SD rat mesenchymal stem cell growth medium) was added per well. Twenty-four hours after transduction, the medium was changed to conventional medium. Forty-eight hours after transduction, subcultured MSCs^ATP7B^ were passaged at a split ratio of 1∶3. Second-or-third passage MSCs^ATP7B^ were harvested for transplantation. For additional cell culture studies, total protein was extracted from MSCs^ATP7B^ five days after transduction for analysis of ATP7B levels by Western Blot. Protein extracted from BM-MSCs transduced with pRRL.PPT.SF.i2GFPpre was used as negative control.

### Preconditioning

To determine which preconditioning was optimal for stimulating proliferation of transplanted cells, LEC rat livers underwent ischemia-reperfusion (IRP) and/or radiation (RT) prior to cell transplantation. For the RT procedure, LEC rats were anesthetized with 10% chloral hydrate (3 mL/kg body weight) by intraperitoneal injection. Four days before transplantation, the whole liver received a single dose of 50 Gy at a dose rate of 320 cGy/min using a RS-2000 x-ray Biological Irradiator (Rad Source Technologies, Inc., Suwanee, GA, USA) as described previously [29]. For IRP, the left portal vein branch was isolated and occluded with a hemostatic clip for 45 min as described previously [30]. Immediately after release of the clip, MSCs^ATP7B^ were injected into the liver via the portal vein.

### Cell Transplantation

Ninety-two-month old LEC rats were divided into 5 groups of 18 animals each: a) saline; b) MSCs^ATP7B^; c) IRP + MSCs^ATP7B^ (IRP group); d) RT + MSCs^ATP7B^ (RT group); e) IRP + RT + MSCs^ATP7B^ (IRP-plus-RT group). Before transplantation, 2×10^7^ MSCs^ATP7B^ were resuspended in 0.5 mL PBS (cell viability was assessed by trypan blue dye exclusion assay), then transfused into the portal vein with a 30-gauge needle for over 2 min. Rats were sacrificed 4, 12 and 24 weeks later. At each time point, fresh liver, brain, and kidney tissues, and blood were obtained for analysis.

### Biochemical Analysis and Histopathology

Blood samples at each time point were collected in heparin-containing tubes and centrifuged at 5000×g for 10 min. Levels of alanine aminotransferase (ALT) and aspartate aminotransferase (AST) were measured with a biochemical analyzer (Hitachi Co. Ltd., Tokyo, Japan). Formalin-fixed liver sections (5 µm) were stained with hematoxylin and eosin (HE) and Masson's trichrome (MT). The fibrotic area by MT staining was quantified by image analysis (Image-Pro Plus 6.0; Media Cybernetics, Bethesda, MD) and the histological features were independently assessed by two pathologists who were blinded to other details of the experiments.

### Immunofluorescence

To confirm that injected MSCs^ATP7B^ had differentiated into normal hepatocytes, GFP/CK-18 double fluorescence intensity was assessed using a previously described protocol [31]. Briefly, liver tissues were embedded in tissue freezing medium (Tissue-Tek OCT Compound, Sakura Finetek, Torrance, CA, USA) and frozen at −80°C. Frozen sections were cut at 4 µm, fixed in cold acetone and then blocked in bovine serum albumin (BSA). Anti-CK-18 rabbit monoclonal antibody (Abcam, Cambridge, MA, USA) and anti-GFP mouse monoclonal antibody (Abmart, Shanghai, China) diluted, respectively, to 1∶100 and 1∶500 in PBS containing 1% BSA were applied to the blocked sections for 16 hours at 4°C. Negative controls were incubated with PBS containing 1% BSA instead of primary antibody. After washing in PBS, FITC-conjugated donkey anti-rabbit IgG (1∶1000, Abcam, Cambridge, MA, USA) and PE-conjugated goat anti-mouse IgG (1∶400, Santa Cruz, CA, USA) were applied for 1 hour at room temperature.

### Real-time Quantitative Polymerase Chain Reaction (RT-PCR)

Total RNA was extracted from frozen liver tissues using Trizol solution (Invitrogen Life Technologies, Carlsbad, CA, USA) and then treated with RNase-free DNase. mRNA was reverse transcribed using a commercially available kit (Perfect Real Time, SYBR PrimeScriP TaKaRa, Shiga, Japan). The RT-PCR analysis was carried out with the following primers: ATP7B (F: 5′-GCCAGCATTGCAGAAGGAAAG-3′ and R: 5′ -TGATAAGTGATGACGGCCTCT-3′) and cDNAs for 40 cycles of 95°C for 5 min, 95°C for 30 s, 60°C for 30 s and 72°C for 30 s according to the manufacturer's protocol using the ABI Prism 7000 Sequence Detection system (Applied Biosystems, Tokyo, Japan). Ct values produced by ATP7B primers were normalized to the expression of the ß-actin housekeeping gene (2^−ΔΔCt^ method).

### Western Blot Analysis

Western Blots were performed with slight modifications to a previous protocol [32]. Cell lysates were prepared by incubation in cold lysis buffer containing 25 mmol/L Tris-Cl (pH 7.5), and 5 mmol/L EDTA 1% SDS; the samples were then mixed with loading buffer and boiled at 100°C for 5 min. The concentration of protein was determined using the bicinchoninic acid (BCA) protein assay kit (Beyotime Institute of Biotechnology, Shanghai, China). Samples were subjected to electrophoresis in SDS-PAGE and transferred to polyvinylidene fluoride membranes. Membranes were blocked in 5% blocking buffer for 2 hours at room temperature, and incubated with the primary antibodies, overnight at 4°C. After washing in TBS with 0.1% Tween-20, membranes were incubated with secondary antibodies for 1 hour at room temperature. The primary antibodies used in this study were rabbit monoclonal anti-ATP7B antibody (1∶3000 dilution, Abcam, Cambridge, MA, USA) and rabbit polyclonal anti-β-tubulin antibody (1∶1000 dilution, Cell Signaling Technology, Danvers, MA, USA).

### Copper Measurement

Fresh liver samples at each time-point were desiccated under vacuum at 65°C for 16 hours, then solubilized in nitric acid. Levels of copper were determined by atomic absorption spectroscopy (AAS) at the Center of Modern Analysis, Nanjing University, according to a protocol described previously [33]. Results were expressed as µg/g dry weight of tissue.

### Serum ceruloplasmin analysis

Serum ceruloplasmin at each time-point was determined using the method of Malhi et al. [33]. Results were expressed as mg/dL.

### Statistical analysis

All data were expressed as mean ± standard deviation, and were compared by ANOVA followed by Student-Newman-Keuls (SNK) post-hoc analysis using GraphPad Prism 5 software. Differences were considered significant at *p* values <0.05.

## Results

Ninety healthy two month old LEC rats were used in this research. They were randomly divided into 5 groups: group a were injected with saline, group b were injected with MSCs^ATP7B^, groups c, d and e had livers preconditioned with ischemia-reperfusion (IRP), radiation (RT) and ischemia-reperfusion plus radiation (IRP-plus-RT), respectively before injection with MSCs^ATP7B^.

### Characterization of BM-MSCs derived from LEC rat bone marrow

**Figure 1:**
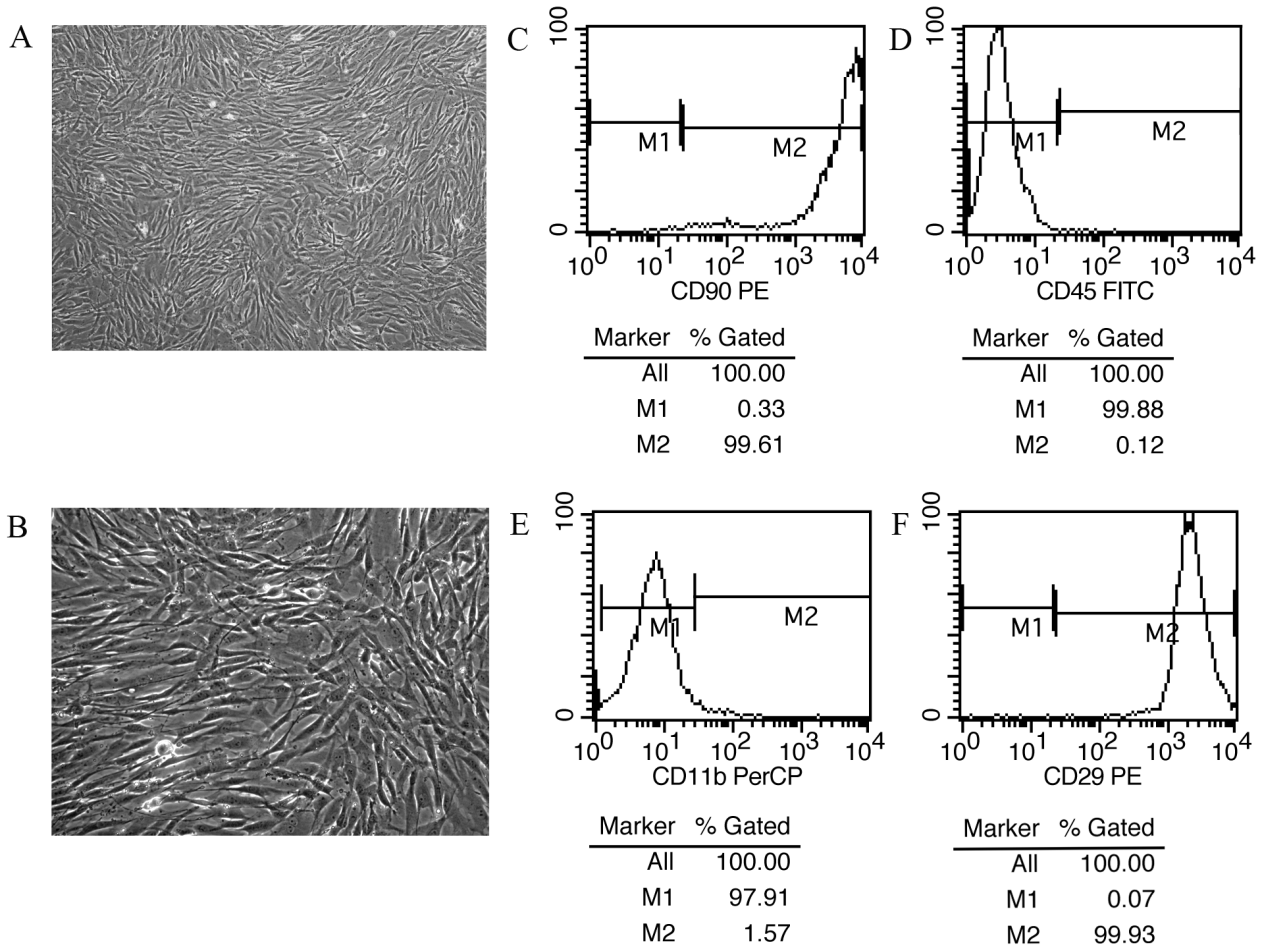
Morphology and characterization of BM-MSCs. (A, B): The morphology of BM-MSCs of passage 5 (×100 magnification [A] and ×200 magnification [B]). The BM-MSCs have a fibroblast-like, spindle-shaped morphology. (C–F): Immunophenotype of BM-MSCs. BM-MSCs of passage 5 were marked with antibodies specific for positive antigen (CD 90 [C] and CD 29 [F]) or negative controls (CD45 [D] and CD11b/c [E]), and analyzed by flow cytometry.

BM-MSCs were isolated from rat bone-marrow mononuclear cell fractions by the direct plastic adherence method. BM-MSCs were observed to be spindle-shaped, fibroblast-like (figures 1A–B), and maintained their undifferentiated status.

The surface markers of BM-MSCs were identified by flow cytometry. Over 99% of the isolated BM-MSCs expressed CD29 and CD90, but not CD11b/c and CD45 (figures 1C–F).

### Transduction of BM-MSCs in cell culture

**Figure 2:**
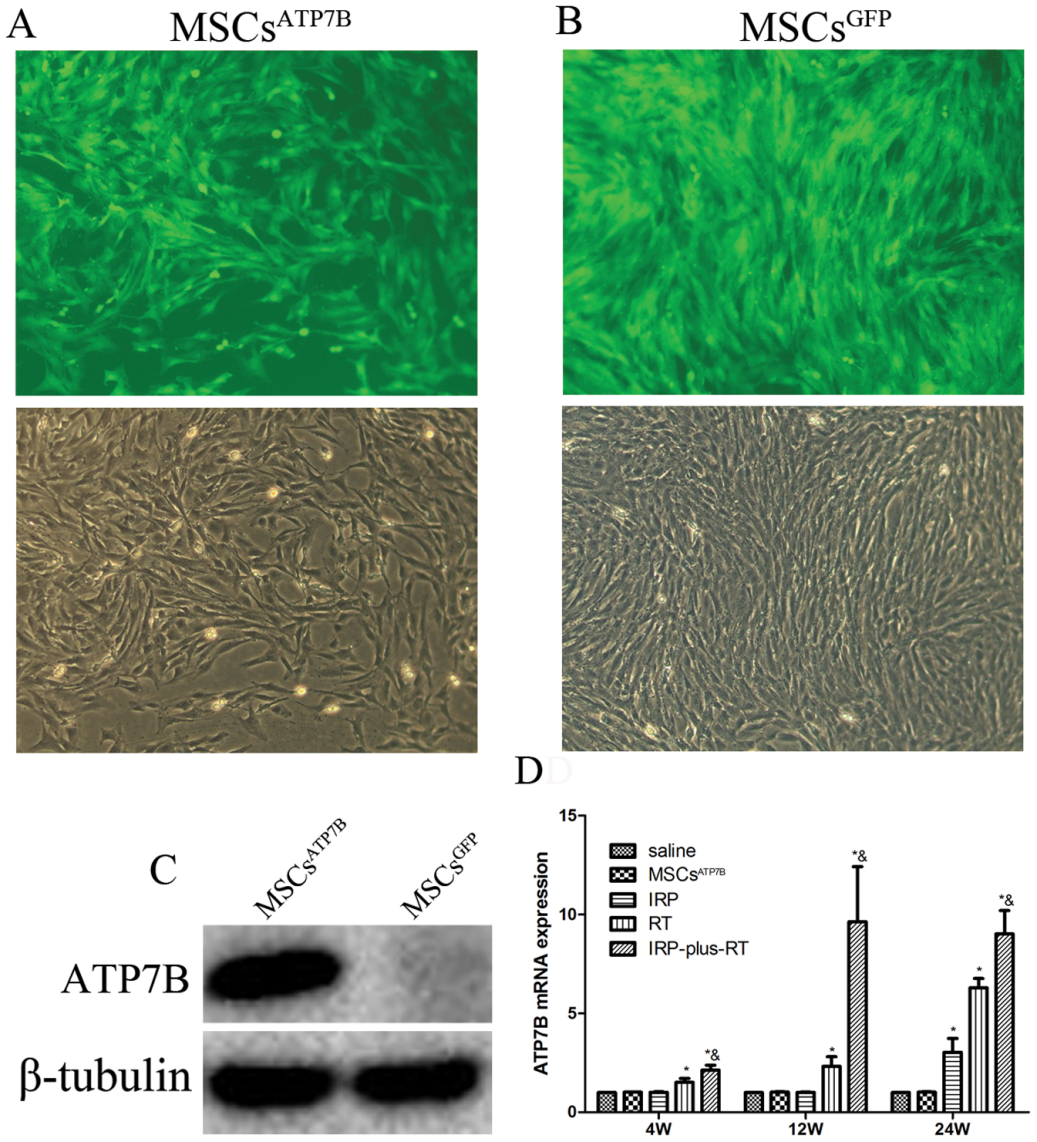
Transfection efficacy, expression of ATP7B post-transfection and post-transplantation. (A) Transfection efficacy of pRRL.PPT.SF.ATP7B.i2GFPBsd.pre in BM-MSCs. (B) Transfection efficacy of pRRL.PPT.SF.i2GFPpre in BM-MSCs. Transfected cells were observed under both a fluorescence microscope and a regular microscope (×100); (C) Expression of ATP7B in BM-MSCs post-transfection assessed by Western Blot. (D) Expression of ATP7B in each group at each time-point following transplantation *in vivo*. ^*^ *p*<0.05 compared with the saline group, ^&^ *p*<0.05 compared with IRP and RT group, respectively.

At 48-hours post-transduction, nearly 100% of cells were transduced (figures 2A and 2B). To analyze the functional activity of BM-MSCs, 5 days after transduction, total proteins extracted from MSCs^ATP7B^ and MSCs^GFP^ were analyzed by Western Blots. The protein levels of ATP7B were markedly enhanced in MSCs^ATP7B^ (figure 2C).

### Expression of ATP7B, post-transplantation, as a function of time

Liver ATP7B expression was measured by RT-PCR at 4, 12, and 24 weeks post-transplantation. As shown in Figure 2D, at all three time points, the ATP7B expression of both the RT group and the IRP-plus-RT group were significantly higher than the saline group (for all, *p*<0.05). The same comparison between the IRP group and the saline group showed a significant difference only at 24 weeks post-transplantation. It was also found that ATP7B expression in the IRP-plus-RT group was markedly higher than that in either the IRP or the RT group at each time point post-transplantation (*p*<0.05, for all).

### Engraftment and repopulation of MSCs^ATP7B^ after transplantation

**Figure 3:**
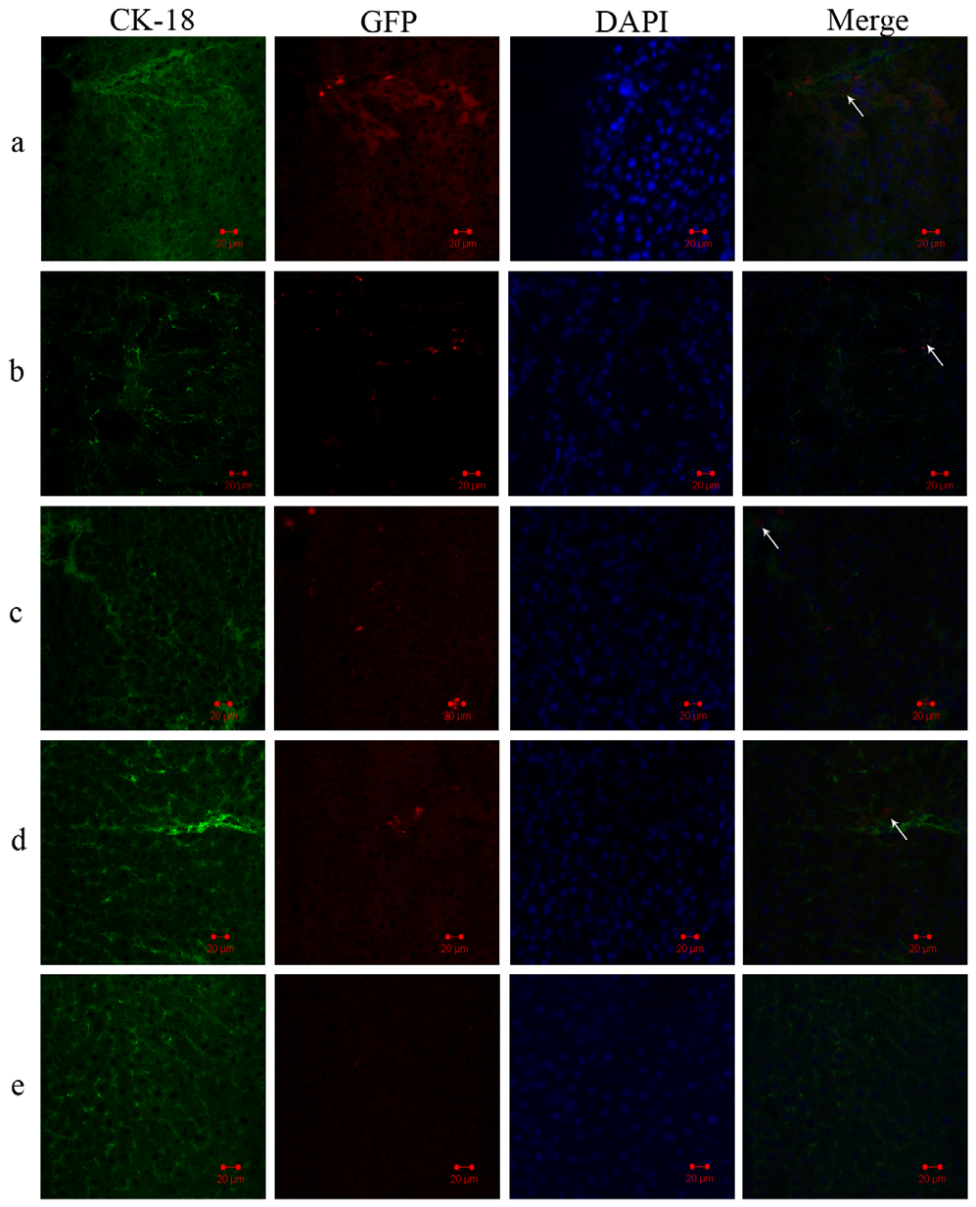
Migration and population of transplanted BM-MSCs *in vivo*. Expression of CK-18 in GFP labeled BM-MSCs engrafted in liver tissues 4 weeks after transplantation; detection by immunofluorescence histochemistry. (DAPI, 4′,6-diamidino-2-phenylindole). The collected figure illustrates hepatocytes differentiated from transplanted MSCs^ATP7B^: (a) IRP-plus-RT group, (b) RT group, (c) IRP group, (d) MSCs^ATP7B^ group, (e) saline group.

To track hepatocytes that had differentiated from MSCs^ATP7B^, a red-fluorescent second antibody was used to detect GFP transduced into MSCs as a marker of mature hepatocytes. A green-fluorescent second antibody was used to mark CK-18 of the hepatocytes. GFP/CK-18 positive cells (figure 3) were detected around both hepatic sinusoids and within the parenchyma of recipient livers of the IRP-plus-RT group and the RT group at 4 weeks post-transplantation. These cells remained present in recipient livers through 24 weeks following transplantation. However, there were few GFP/CK-18 positive cells in the livers of either the IRP group or the MSCs^ATP7B^ group and no GFP-positive cells in the liver of the saline group were observed at any time point post-transplantation. No GFP fluorescence intensity was detected in other organs such as brain and kidney (data not shown).

### Protective effect of transplantation of MSCs^ATP7B^ on liver histopathology

**Figure 4:**
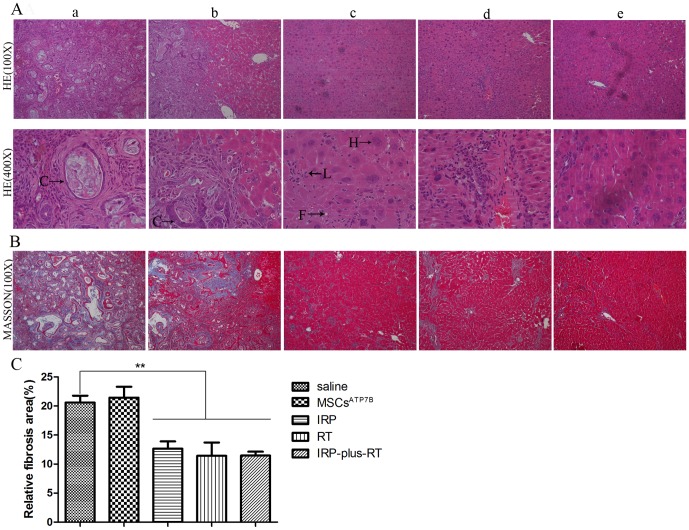
Hematoxylin and eosin and Masson's trichrome staining of liver sections 24 weeks post-transplantation. A. H&E staining, (a, b): Typical histological finding of cholangiocarcinoma seen in the liver of saline and MSCs^ATP7B^ groups. (c–e): Livers of IRP, RT, and IRP-plus-RT rats, respectively. The typical histological finding of cholangiocarcinoma is absent, but the histological finding of chronic hepatitis is present. Enlarged nuclei and fatty changes are visible in some of the hepatocytes; lymphocytes are irregularly distributed among hepatocytes. (C: cholangiocarcinoma; H: regenerated hepatocytes; L: lymphocytes; F: fine fatty droplets); B. MT staining. C. Quantitation of MT staining. The liver fibrosis of IRP, RT, and IRP-plus-RT groups are compared with the saline group, respectively; IRP and RT groups are also compared with IRP-plus-RT group, respectively. ^**^ *p*<0.01.

HE staining showed that cholangiocarcinomas were present in the livers of both the saline (4/6) and MSCs^ATP7B^ (5/6) groups 24 weeks post-transplantation, while only chronic hepatitis was observed in the IRP, RT and IRP-plus-RT groups (figure 4A). Moreover, as shown in Figure 4B–C, compared with saline group, a significant decrease in liver fibrosis were observed in the IRP, RT and IRP-plus-RT groups (12.66±1.26% for IRP group, 11.42±2.30% for RT group and 11.46±0.68% for IRP-plus-RT group versus 20.60±1.19% for saline group, *p*<0.01, for all). However, there were no significant differences in liver fibrosis between the IRP-plus-RT group and IRP or RT groups, both *p*>0.05.

### Liver copper concentration

Liver copper concentration (µg/g dry weight tissue) was determined 4, 12, and 24 weeks following MSCs^ATP7B^ transplantation. As shown in Table 1, liver copper concentration increased from 466±39 µg/g to 1240±106 µg/g in the saline group, and 454±42 µg/g to 1246±100 µg/g in the MSCs^ATP7B^ group. Liver copper concentrations increased from 453±36 µg/g to 758±92 µg/g in the IRP group, 308±23 µg/g to 450±33 µg/g in the RT group and 229±20 µg/g to 341±28 µg/g in the IRP-plus-RT group. There were no significant differences in liver copper concentrations between the saline group and MSCs^ATP7B^ group (*p*>0.05) at any time-point. Twenty-four weeks after transplantation, liver copper concentrations in the IRP group were significantly lower than in the saline group (*p*<0.05). Moreover, liver copper concentration of the RT group and the IRP-plus-RT group was significantly lower than that of the saline group at all time-points (*p*<0.05, for all). Additionally, rats preconditioned with IRP-plus-RT demonstrated decreased liver copper concentration compared with rats preconditioned with IRP alone or RT alone at each time-point post-transplantation (*p*<0.05, respectively). No significant differences in the copper concentration of brain and kidney were found between treatment groups and the saline group at all time points (data not shown).

**Table 1:** Changes in Hepatic Copper Content of LEC Rats as a Function of Time after MSC^ATP7B^ Transplantation (µg/g dry weight tissue).

Preconditioning	n	4W	12W	24W
Saline	6	466±39	907±20	1240±106
MSCs^ATP7B^	6	454±42	890±30	1246±100
IRP	6	453±36	893±14	758±92*
RT	6	308±23*	646±17*	450±33*
IRP-plus-RT	6	229±20* ^&^	509±24* ^&^	341±28* ^&^

IRP: Ischemia Reperfusion, RT: radiation.

*p<0.05, compared with the saline group.

& *p*<0.05, compared with IRP and RT groups, respectively.

### Serum ceruloplasmin analysis

Serum ceruloplasmin levels (mg/dL) were compared between treatment and the saline groups as well as between rats preconditioned with IRP-plus-RT and rats preconditioned with either IRP alone or RT alone at each time-point following MSCs^ATP7B^ transplantation. The results are shown in table 2. Briefly, serum ceruloplasmin was decreased from 7.1±1.6 mg/dL to 6.1±1.5 mg/dL in the saline group, and from 8.5±1.9 mg/dL to 7.0±3.4 mg/dL in MSCs^ATP7B^ group. Serum ceruloplasmin increased from 7.7±2.6 mg/dL to 16.2±2.0 mg/dL in the IRP group. Serum ceruloplasmin increased from 15.2±2.4 mg/dL to 21.5±2.0 mg/dL in the RT group, and from 19.3±3.2 mg/dL to 25.0±2.2 mg/dL in the IRP-plus-RT group. Rats treated with IRP-plus-RT and RT alone showed a higher serum ceruloplasmin level than rats in the saline group at all time points (*p*<0.05, for all). In the IRP group, serum ceruloplasmin levels were only higher than the saline group at 24 weeks following MSCs^ATP7B^ transplantation (*p*<0.05). In the treated groups, serum ceruloplasmin levels in rats treated with IRP-plus-RT were higher than those in rats treated with IRP alone or RT alone at each time-point (*p*<0.05, for all).

**Table 2:** Serum Ceruloplasmin Levels in LEC Rats 4, 12, and 24 weeks after MSCs^ATP7B^ Transplantation (mg/dL).

Preconditioning	n	4W	12W	24W
Saline	6	7.1±1.6	6.8±3.0	6.1±1.5
MSCs^ATP7B^	6	8.5±1.9	7.6±1.9	7.0±3.4
IRP	6	7.7±2.6	9.3±3.0	16.2±2.0*
RT	6	15.2±2.4*	11.5±2.2*	21.5±2.0*
IRP-plus-RT	6	19.3±3.2* ^&^	17.0±2.8* ^&^	25.0±2.2* ^&^

IRP: Ischemia Reperfusion, RT: radiation.

*p<0.05, compared with the saline group.

& *p*<0.05, compared with IRP group and RT group, respectively.

### Effect of MSCs^ATP7B^ transplantation on ALT and AST levels

**Figure 5:**
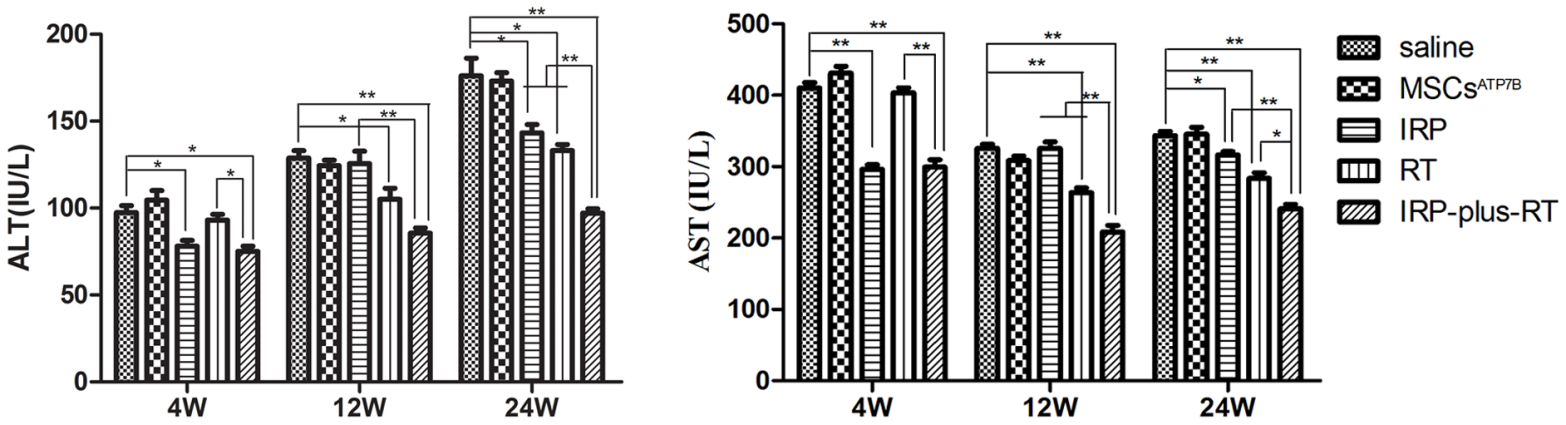
Effect of BM-MSCs on serum biomarkers. Values are expressed as mean ± SD. For ALT and AST levels at each time point, IRP, RT, and IRP-plus-RT groups are compared with the saline group, respectively; IRP and RT groups are also compared with IRP-plus-RT group, respectively. ^*^ *p*<0.05, ^**^ *p*<0.01.

Serum ALT and AST levels were analyzed at each time-point following MSCs^ATP7B^ transplantation, in order to compare the recovery of liver damage in the rats of different groups. As shown in figure 5, 4 weeks after transplantation, a significant reduction in ALT and AST levels was found in the IRP group and the IRP-plus-RT group compared to the saline group (for ALT, 19.8% and 22.9%, both *p*<0.05; for AST, 27.6% and 27%, both *p*<0.01). The levels of ALT and AST in the IRP-plus-RT group were significantly lower than those in the RT group (for ALT, 75±5.29 U/L versus 93±6 U/L, *p*<0.05; for AST, 299.3±18.45 U/L versus 403.3±12.66 U/L, *p*<0.01). At 12 weeks post-transplantation, the ALT and AST levels of the RT group and the IRP-plus-RT group were significantly decreased compared to the saline group (for ALT, 18.4% and 33.4%, *p*<0.05 or 0.01; for AST, 19.2% and 35.9%, both *p*<0.01); and compared with the IRP group, both ALT and AST levels were significant decreased in the IRP-plus-RT group (for ALT, 31.8%, for AST, 35.9%, both *p*<0.01). There was no significant difference in ALT levels between the RT group and the IRP-plus-RT group (*p*>0.05). There was a significant decrease in the AST levels of the IRP-plus-RT group (20.7%, *p*<0.01) compared with those of the RT group. In contrast, 24 weeks post-transplantation, the levels of ALT and AST in the IRP group, the RT group, and the IRP-plus-RT group were all significantly decreased compared to those of the saline group (for ALT, 18.6%, 24.4%, and 44.9%; for AST, 7.9%, 17.4%, and 29.8%, compared with IRP and RT group, *p*<0.05; compared with IRP-plus-RT group, *p*<0.01). Furthermore, ALT levels were significantly lower in the IRP-plus-RT group compared to both IRP and RT groups (32.3% and 27%, *p*<0.01, respectively). The same comparison for results for AST level showed 23.8% and 15% differences, (*p*<0.01 and *p*<0.05, respectively).

## Discussion

In recent years, LEC rats and Atp7b^-/-^ mice have been shown to be suitable models for gene therapy of WD [34]. Terada *et al.* [35] administered recombinant adenovirus containing WND (ATP7B) cDNA into LEC rats by tail vein injection, and showed that the holoceruloplasmin levels were partially restored within two weeks. However, the expression of the WND gene tended to disappear after two weeks. Moreover, adenovirus vectors have a relatively strong toxicity and can induce an immune response. To overcome this shortcoming, Merle *et al*. rescued LEC rats with modified lentiviral vectors. However, quantitative immunofluorescence analysis of liver tissue sections showed a lower percentage of ATP7B-positive hepatocytes 24 weeks after treatment when compared to 2 weeks after treatment [36]. Two problems remained unresolved in this research. First, implanted hepatocytes could not correct the liver injury caused by the disorder of copper metabolism. Second, although several studies have confirmed that hepatocyte transplantation is effective for correcting the copper metabolism of LEC rats [37]–[40], the inability of transplanted cells to proliferate in normal liver prevents cell therapy from being clinically useful. In the current experiments, we obtained prolonged expression of ATP7B which is consistent with our previous results using BM-MSCs and lentiviral vectors in gene therapy [41], [42].

Oxidative hepatic DNA damage induced by IRP or RT is known to impair the survival of native cells, and promote proliferation of transplanted cells. Malhi *et al.* [29] preconditioned F344 rats with whole liver radiation and warm ischemia-reperfusion followed by intrasplenic transplantation of syngeneic F344 rat hepatocytes, and found that the proliferation of transplanted cells in the animals treated with IRP-plus-RT was far more successful than that in control animals as well as in animals preconditioned with either IRP or RT alone. A similar study was also conducted in LEC rats, and showed that the resulting long-term reversal of copper toxicity in the animals preconditioned with IRP-plus-RT occurred because of superior engraftment and proliferation [33]. Consistent with these two results, we found that ATP7B expression lasted up to 24 weeks post-transplantation, and that the copper concentration, serum ceruloplasmin, and liver functions of the IRP-plus-RT group were obviously improved compared to those of the saline, IRP, or RT groups. The possible mechanisms by which IRP and RT promote engraftment and proliferation may lie in the selective pressure of liver damage. Selective pressure in the form of liver injury has been proven to be required for donor cell engraftment of the liver [43]. However, the limitations of IRP and/or RT preconditioning such as acute hepatitis cannot be ignored, although such preconditioning was well-tolerated by all animals. Malhi *et al.* [29] have confirmed that acute liver injury could be produced by such strategies.

Recently, Sauer *et al.* [44] mimicked high hepatic copper conditions in cell culture and found that ATP7B overexpression confers an important viability and selective advantage to cells in toxic copper microenvironments. Furthermore, previous researchers have shown that human MSCs can integrate into the livers of rats and mice and differentiate into functional hepatocytes [18], [45], suggesting that ATP7B-transduced MSCs might be suitable for therapy of WD. Also, *in vivo* application of ATP7B encoding viral vectors to the liver was found to restore copper metabolism and holoceruloplasmin synthesis in LEC rats [35], [46]. This is the best evidence that the expression of ATP7B is necessary for therapy of WD. However, such experimental conditions are not directly applicable to WD patients in the clinic, our present study included. Reasons for this may be the following: (1) the safety of the viral vectors in humans is still unknown; (2) infusion of cells into the portal vein is known to cause sinusoidal occlusion and portal hypertension; and (3) in our present study, we rescued the LEC rats with MSCs^ATP7B^ when the rats were 8 weeks old. LEC rats have no signs of WD at this time point. In other words, the treatment was preemptive, but not proof of disease improvement. However, in clinical situations, the livers of WD patients are often cirrhotic when WD symptoms appear. Treatment of such patients by stem cell transplantation may not be very effective due to the damaged hepatic architecture. Therefore, further studies are needed to determine the timing and the best infusion approach for stem cell transplantation of WD patients.

In summary, transplantation of ATP7B-transduced bone marrow mesenchymal stem cells decreases copper overload in rats.
